# Towards an Integrative Taxonomy of Social-Emotional Competences

**DOI:** 10.3389/fpsyg.2021.515313

**Published:** 2021-03-11

**Authors:** Ingrid Schoon

**Affiliations:** Institute of Education, University College London, London, United Kingdom

**Keywords:** social-emotional competences, integrative taxonomy, conceptualization, core domains, manifestations, self-determination

## Abstract

Social-emotional competences are critical for positive development and significantly predict educational and occupational attainment, health, and well-being. There is however a lack of consensus about the number of core competences, and how these are defined and operationalized. This divergence in approach challenges future research as well as the scientific usefulness of the construct. In an effort to create an integrative framework, this focused review evaluates different approaches of conceptualizing and assessing social-emotional competences. Building on shared conceptions, an integrative taxonomy “DOMASEC” is introduced, specifying core domains and manifestations of social-emotional competences that bridge across frameworks focusing on social and emotional learning, personality traits (such as the Big Five) and self-determination theory. Core domains include intrapersonal, interpersonal and task-oriented competencies, differentiating between affective, cognitive, and behavioral manifestations of competences across these domains. It is argued that the integrative taxonomy facilitates the conceptual specification of key constructs, that it helps to better organize the multitude of terms and definitions used, and to guide the conceptualization and operationalization of social-emotional competences and their various facets.

## Introduction

Social and emotional competences are increasingly recognized as important predictors of valued life outcomes, such as educational and occupational attainment, health and wellbeing (OECD, 2015). They are considered to be essential in tackling key developmental tasks, such as succeeding in education, in the workplace, in social relationships, and life in general (Gutman and Schoon, 2016). Moreover, social-emotional competences are relevant in enabling individuals exposed to numerous risk factors, such as poverty or family adversity, to succeed against the odds (Elias and Haynes, 2008; Domitrovich et al., 2017; Schoon and Lyons-Amos, 2017). Indeed, they are thought to be as important as cognitive competences in shaping one's life (Heckman and Kautz, 2012). There is however no consensus yet about the number of core competences, and how these are defined and operationalized. The lack of shared definitions and approaches in assessment poses challenges to future empirical research and raises questions regarding the usefulness of social-emotional competences as a scientific construct (Pellegrino and Hilton, 2012). To advance the field, there is thus a need to more clearly delineate and distinguish core domains and manifestations of social-emotional competences. The aim of this focused review is to introduce an integrative framework for the study of social-emotional competences, building on shared conceptions in the field. First, a summary of the overarching terms and shared attributes underlying different conceptualizations of social-emotional competences is provided. Next, an integrative taxonomy of core domains and manifestations of social-emotional competences is introduced, highlighting in particular the role of the individual as an agent in their own development. Then different approaches for assessing the different domain and manifestations of social-emotional competences are discussed and suggestions for possible avenues for future research are made.

## Conceptualizing Social-Emotional Competences

The conceptualization and measurement of social-emotional competences is not a straightforward task, because the term refers to a set of more specifically delineated competences. The notion of social-emotional competences is generally used as an umbrella term, referring to a range of capabilities that enable individuals to express, regulate and understand their thoughts, emotions, behaviors in every-day situations and interactions with others, and to adjust to changing conditions. Moreover, social-emotional competences are known under different terms, such as “non-cognitive,” “character” or “soft” skills, contrasting them to the more directly assessable cognitive competences (Duckworth and Yeager, 2015; Abrahams et al., 2019). The terms skill and competence are sometimes used interchangeably, although there is potentially a difference in scope, with competence being the broader term, incorporating a set of skills (National Research Council, 2012; OECD, 2015). In empirical research, approaches to operationalize social-emotional competences and skills vary across laboratories and across disciplines.

There is considerable variability in the number and nature of the social-emotional competences included in different approaches and frameworks (Abrahams et al., 2019; Jones et al., 2019). Many authors differentiate between intrapersonal competences (such as self-control and emotion regulation) and interpersonal competences (such as perspective taking, and relationship skills) enabling effective functioning and interactions with others (Malti and Noam, 2016; Domitrovich et al., 2017). Some use the Big Five personality dimensions as a guidepost (De Fruyt et al., 2015; Abrahams et al., 2019), while others focus on distinct competences or skills, such as the ability for self-regulation (Blair, 2002; Moffitt et al., 2011), or goal-directed efforts such as grit or persistence (Duckworth et al., 2007). In addition, there are approaches to bundle different indicators into a composite, not differentiating between distinct dimensions (Liu, 2019).

Efforts to specify the communalities of social-emotional competences can be grouped into three major approaches: First, classifications related to the development of screening instruments such as the Achenbach System of Empirically-Based Assessment (Achenbach, 2019), or the Strengths and Difficulties Questionnaire (Goodman et al., 2000) derived from clinical observations. These instruments have strong psychometric properties and are used for the identification of emotional and behavioral adjustment in general population and clinical samples. They are however, mostly focused on the identification of adjustment problems instead of strengths or competences.

Second, conceptual approaches adopted by the Collaborative for Academic, Social Emotional Learning (CASEL) aim to enable the development of core social and emotional competencies that contribute to children's school success and life outcomes. Rooted in theories of progressive education, transactional models of human development, and the emotional intelligence literature (Osher et al., 2016), fundamental goals of the CASEL framework are to promote positive learning environments that are supportive and engaging and to foster the development of five interrelated sets of competencies comprising intrapersonal skills (such as self-awareness, self-management), interpersonal skills (social awareness, relationship skills), and task performance (responsible decision-making). These core competences are considered to enable student's capacity to integrate emotion, reflection and behavior across everyday personal and social challenges (Durlak et al., 2015). A major aspect of the SEL approach is its developmental-contextual focus, accounting for developmental processes involved in socio-emotional learning and the associated empirical evidence confirming the role of interventions and contextual influences in promoting the development of key skills and competences (Durlak et al., 2011; Jones et al., 2019). Notable gaps in the SEL research framework are the need for practical, reliable and valid assessments of specific SEL skills, and the need to clarify terminology and align language and frameworks (Osher et al., 2016). Yet, there have been recent advances in the development of valid and reliable assessment scales, and the factor structure of SEL framework could be confirmed (Mantz et al., 2018; Gresham et al., 2020). This evidence is mirrored in findings confirming the factor structure underlying the assessment of emotional intelligence along indicators of self-awareness, self-management, social awareness, relationship management, and problem solving (Boyatzis, 2018).

Third, data-driven efforts such as the use of psycholexical analysis aim to group different descriptions of personality into a smaller number of overarching constructs. Resulting frameworks, such as the Big Five model, reflect personality traits comprising self-management (conscientiousness), engaging with others (extraversion), collaboration with others (agreeableness), negative emotion regulation (neuroticism), and open-mindedness (openness to experiences) (John et al., 2008; Abrahams et al., 2019). Terms such as personality traits are used refer to relative stable dispositions that account for consistencies in behavior, thought and feeling across situations and over time (Costa et al., 2019). There is however also evidence of developmental processes (Caspi et al., 2005), suggesting that personality traits are both stable and malleable (Damian et al., 2019), pointing to the role of environmental factors, such as life events (Bleidorn et al., 2018), as well as interventions (Roberts et al., 2017) to contribute to that change. The underlying five factor personality structure, derived from the exploration of English lexical personality terms, has been confirmed across many cultures (McCrae et al., 2005). However, approaches using indigenous lexical study could not fully replicate the five factor personality structure (De Raad et al., 2010). Moreover, indigenous lexical studies conducted in Asia identified an additional factor of interpersonal relatedness (Cheung et al., 2008, 2011) which is not represented in the Big Five Framework. In addition, the Big Five Framework as such does not account for competences reflecting self-awareness, such as self-concepts, or the ability to correctly understand the social cues of others (John et al., 2008), and a broader approach is needed to comprehensively classify key competences.

Advancing the field is however hampered by the situation, that despite considerable overlap and similarities in the constructs derived from these different approaches. The conceptualization of social and emotional competences has been afflicted by what some authors call the “jingle and jangle fallacy” (Borghans et al., 2008; Jones et al., 2016). The “jingle fallacy” refers to the use of a same term for different constructs, while the “jangle fallacy” refers to the use of different terms for similar constructs. Moreover, variability in terms can be justified due to processes of developmental maturation and change over time. For example, initially reactive forms of self-control in children develop into more intentional and flexible forms of self-regulation (Montroy et al., 2016; Booth et al., 2018). There are thus multiple challenges in moving forward toward a consensual definition, including differences in terminology (which can vary according to discipline or field of study), differences in focus, and aspects of developmental change.

Despite differences in terminology and assessment, there is agreement in that social and -emotional competences refer to individual-level capabilities involved in understanding and accepting oneself, in negotiating every-day situations and interactions with others, to deal with challenges and to adjust to changing conditions. Social-emotional competences (1) are conceptually different from academic abilities and subject-matter achievement; (2) originate through reciprocal interactions between biological predispositions and contextual influences; (3) develop progressively as children mature; (4) are shaped through socialization experiences and learning (in formal and informal settings) and are thus understood to be malleable and responsive to intervention; (5) are manifested in more or less consistent patterns of thoughts, feelings and behaviors, although they can vary across contexts and over time; (6) are dependent on situational factors for their expression; and (7) are crucial to success in school, across a wide range of socio-economic outcomes in later life, as well as health and wellbeing (Blair, 2002; De Fruyt et al., 2015; Duckworth and Yeager, 2015; Gutman and Schoon, 2016; Domitrovich et al., 2017; Bailey et al., 2019).

## Toward An Integrative Taxonomy of Social-Emotional Competences

Previous efforts to create an integrated taxonomy of social-emotional competences argued for the alignment of all existing constructs within a single existing framework, such as the Big Five (Abrahams et al., 2019), which would help to reduce complexity and generate a common language. However, as argued above, the Big Five Framework is not broad nor specific enough to capture competences relevant across different cultural contexts. The evidence suggests that models with fewer factors are more robust, in particular when replicated across different cultural contexts (De Raad et al., 2010). In addition, some have argued that it is necessary to differentiate between skills and traits (Duckworth and Yeager, 2015) and to draw a clear conceptual distinction between traits that reflect what someone tends to do, and capacities that reflect what someone is capable of doing (Soto et al., 2020). Other integrative efforts to create new frameworks are based on a review of existing research on social-emotional competences, including taxonomies derived from Social and Emotional Learning (SEL) and Big Five frameworks (National Research Council, 2012; OECD, 2015). For example, the framework developed by the National Research Council (National Research Council, 2012) identified three core 21st century skill clusters comprising intrapersonal, interpersonal, and cognitive competences—the latter also including information literacy, i.e., using knowledge effectively. The OECD Definition and Selection of key Competences (DESECO) Framework (which was developed in collaboration with a wide range of experts from different academic disciplines, countries and international organizations) also identified three core skill domains, comprising social, emotional as well as cognitive skills including the ability to act autonomously, to interact in socially heterogeneous groups, and to use tools interactively (Rychen and Salganik, 2003). These competences are considered necessary to enable full participation in society, in particularly regarding participation in the work force—with relevance also in developing and transition countries. However, including skills that reflect the effective use of tools or information/knowledge moves these conceptual frameworks beyond the focus of social-emotional core competences.

Focusing on competences commonly found in SEL-focused frameworks, the Harvard-based “Taxonomy Project” aimed to identify areas of overlap and distinction between different personality and SEL-focused frameworks (Berg et al., 2017; Jones et al., 2019). The objective of the Taxonomy Project was not to develop a new framework or privileging one framework over another—but to generate a taxonomy of social-emotional skills designed to link terms across frameworks. This work resulted in the development of an online repository, including a tool (Explore SEL) that connects over 60 conceptual frameworks, illustrating how different social and emotional constructs are related to one another and across disciplines. The taxonomy groups skills into six domains (cognitive, emotional, social, values, perspectives, and self-image/identity) and their domain-specific facets. For example, terms within the emotional domain are grouped into subdomains of empathy/perspective taking, emotional knowledge and expression, and emotional, and behavioral regulation. These domains and subdomains have been empirically identified, yet there is no conceptual specification of them and the coding is described as a work in progress.

### The DOMASEC Classification

Building on this previous work, I propose a two-level taxonomy of key domains and manifestations of social-emotional competences (DOMASEC) which serves to better organize the multiple terms and constructs used in the study of social-emotional competences across disciplines. The DOMASEC model is not intended as a new framework, but as an integrative approach linking across existing frameworks, such as CASEL, the Big Five and others, aligning language with the aim to offer conceptual clarity and to help with the identification and classification of constructs, and where applicable to assess and measure social and emotional competences. The model is guided by developmental-contextual approaches (Bronfenbrenner and Morris, 2006), acknowledging that human development does not take place in a social vacuum and recognizing the bi-directional influences between a developing individual and a changing context that produce continuity and change in individual characteristics over time (Sameroff, 2010). It also builds a bridge to self-determination theories (SDT) (Ryan and Deci, 2017) which emphasize the human need to learn, to extend oneself, and to apply one's talents.

Core domains of the DOMASEC model comprise orientations toward the self (intrapersonal competences), toward others (interpersonal competences), and toward developmental tasks (such as succeeding in education, making decisions about employment, or adapting to changing conditions). The core domains reflect the ways in which individuals perceive themselves, interact with others, and engage with their environment, e.g., the challenges or tasks they encounter, or the goals they set themselves. Together these domains emphasize the role of the individual as an actor, and the need to engage in and to adapt to different and changing challenges and demands over the life course. At the second level, a differentiation is made between the affective, cognitive, and behavioral manifestations of functioning across different domains. Affective manifestations reflect the positive or negative feelings about the self, others, or different tasks. Cognitions indicate the believes, thoughts and knowledge about the self, others, or different tasks, and behavior the manifest conduct and approach.

It is important to take into account different domains and manifestations of social-emotional competences, since some individuals might show effective task-performance and act in correspondence with socially accepted norms and expectations, despite being emotionally unbalanced. Or, they might be well attuned in regulating their interactions with others, but not in concentrating their commitment to specific tasks. The identification of different core domains and manifestations of emotions, thoughts and behaviors within and across these domains is considered necessary to facilitate the conceptual specification of key constructs, directing focus to the most salient aspects of their expression. Considering the multiple domains and manifestations of social-emotional competences enables the assessment of variations in adjustment and the identification of potential competence profiles.

Table 1 gives examples of prototypical competences for each of the manifestations across domains. The taxonomy differentiates variations in emotional response toward the self (such as feelings of self-esteem), toward others (empathy), or toward specific tasks (such interest or valuing them). Moreover it takes into account cognitions or believes about the self (self-concept), about others (perspective taking), or specific tasks (foresight), as well as behavioral manifestations, such as self-regulation, ways of regulating one's interaction with others (cooperation), and efforts to achieve a task or goal. These manifestations change or can vary depending on developmental maturity or different socio-cultural contexts. Nonetheless, the differentiation of the three manifestations facilitates conceptual clarity when trying to classify different constructs, including multi-dimensional constructs, such as grit. Grit comprises passion (an affective aspect) and perseverance, i.e., task-focused behavior directed at the achievement of longer-term goals (Duckworth et al., 2007). Both facets can be captured within the DOMASEC framework, facilitating a better understanding of multiple competences necessary to pursue and achieve a task.

**Table 1:** Domains and manifestations of socio-emotional competences (DOMASEC).

**Domains/manifestations**	**Examples of prototypical competences**	**Examples from other frameworks**			**Basic psychological needs**
		**Big Five**	**CASEL**	**Other (see Explore SEL)**	
**Self-orientation**					**Autonomy**
Affect	Self-esteem	Neuroticism		• Happiness	
Cognition	Self-concept		Self-awareness	• Self-efficacy • Self-reflection • Identity	
Behavior	Self-regulation	Conscientiousness	Self-management	• Self-control • Emotion regulation • Stress regulation	
**Other-orientation**					**Relatedness**
Affect	Empathy			• Compassion	
Cognition	Perspective taking		Social awareness	• Trust • Tolerance • Respect for others	
Behavior	Cooperation	Extraversion agreeableness	Relationship skills	• Connection • Caring • Pro-social behavior • Leadership	
**Task-orientation**					**Competence**
Affect	Value/ Interest			• Zest • Passion	
Cognition	Foresight	Openness	Responsible decision making	• Optimism • Purpose • Inquisitiveness • Imagination/creativity	
Behavior	Task-performance			• Persistence/effort • Initiative • Innovation	

The aim of the DOMASEC taxonomy is to be broad enough to integrate social-emotional competences studied across different disciplines, and to be specific enough to enable the grouping of social-emotional competences according to their core domains and their most central manifestations. In particular, the DOMASEC specification of core domains will facilitate the classification of competences recognized across different fields.

### Integration of Different Frameworks

The DOMASEC taxonomy is not intended to be a grand theory of human development, it rather aims to facilitate the classification of social-emotional competences studied across different disciplines within an integrative framework. One of the central goals of scientific taxonomies is the specification of overarching domains within which large numbers of specific instances can be understood in a simplified way. It is hoped that the DOMASEC framework, as a generally accepted taxonomy, would facilitate the accumulation and communication of empirical findings by offering a standard vocabulary, or nomenclature.

Table 1 illustrates how the DOMASEC model maps onto different frameworks, such as the Big Five, the CASEL constructs, and other competences specified in prominent classification frameworks (see the Explore SEL online tool). For instance, the DOMASEC framework maps onto the Big Five factors (Abrahams et al., 2019; Costa et al., 2019) which can be grouped as aspects of affective responses toward the self (neuroticism); as behavioral orientations toward the self (conscientiousness) or others (extraversion); or a cognitive response toward a task (openness). Agreeableness also reflects an orientation toward others, but is more difficult to allocate, as it involves a more or less even balance of affective, cognitive and behavioral aspects (Wilt and Revelle, 2015). It's defining facets comprise modesty, trust, and empathy (John et al., 2008) which can be considered as reflections of affective (empathy), cognitive (trust) or behavioral (modesty) aspects of other-orientation. Here agreeableness is grouped as a behavioral aspect of other-orientation, given the centrality of the modesty facet across different Big Five frameworks, such as the lexical approach, the NEO-PI-R and the CPI-Big Five. Notably, the DOMASEC model enables the classification of different sub-facets within each of the Big Five factors and facilitates a more differentiated understanding and conceptualization of key competences. Take another example, the construct of openness, which has been defined as “the breadth, depth, originality, and complexity of an individual's *mental and experiential life*.” (John et al., 2008). Within the DOMASEC framework different facets of openness could be grouped as cognitive orientations toward a task, while others might be considered as cognitive orientations toward the self or others. The first decision in the classification process will be the identification of the core domain, i.e., the focus of orientation toward the self, toward others or a task, followed by a consideration of the most salient form of manifestation, i.e., the affective, cognitive, or behavioral expression.

The DOMASEC model also maps onto a range of other frameworks, such the five competence clusters of the CASEL framework (Durlak et al., 2015), differentiating between cognitive and behavorial manifestations of orientations toward the self (self-awareness, self-management), toward others (social awareness, relationship skills), and toward developmental tasks (responsible decision making). In addition, the two-level DOMASEC taxonomy facilitates a clearer distinction between the core domains and associated feelings, cognitions and behaviors, which do not necessarily have to be consistent across the different domains.

In addition, the three DOMASEC domains capture the central dimensions of internalizing (orientation toward the self) and externalizing adjustment problems as well as prosocial behavior (both reflecting orientation toward others) generally assessed in widely used screening instruments (Goodman et al., 2000; Achenbach, 2019). The three DOMASEC domains also emulate the core skill clusters identified in previous research, i.e., intrapersonal, interpersonal, and cognitive competences (National Research Council, 2012; OECD, 2015), yet the focus is on social-emotional competences not including academic or knowledge skills—instead emphasizing task-oriented competences. The three domains of the DOMASEC taxonomy also correspond to the three-component model of virtue or character strength comprising self-control, caring, and inquisitiveness (McGrath et al., 2018). Self-control can be understood to reflect self-orientation, caring as an aspect of other-orientation, and inquisitiveness as an aspect of task-orientation. The three components of virtue show a considerable degree of overlap with the VIA Classification of Strengths and Virtues (McGrath et al., 2018) and also the Big Five Framework. Despite this overlap the classifications of virtues and personality are however not redundant, highlighting the limitations of a global measure of personality aiming to include all potentially important components of that construct (McGrath et al., 2020).

Moreover, the specification of the three core domains builds a bridge to self-determination theory (SDT) (Ryan and Deci, 2017), opening up new dialogues between interlinked fields of inquiry concerned about the study of human development and wellbeing. SDT specifies a set of innate, universal basic psychological needs for experiencing autonomy, relatedness, and competence. The fulfillment of these basic needs is essential for psychological growth and effective functioning. Autonomy refers to the need to manage one's emotions and behavior, to be able to self-determine what to do. Relatedness refers to the need to care about and be cared about by others, and competence refers to the need to contribute to a cause, to feel challenged and being effective. Within self-determination theory the term competence does not refer to an attained skill or capability, but rather is understood as a felt sense of being effective in interactions with the wider environment, to experience opportunities to exercise and express one's capacities (Ryan and Deci, 2017). Within the DOMASEC classification this aspect denotes engagement with the environment, orientation toward the different tasks encountered, and the way individuals approach, adapt to or change their environment to address their needs.

Linking the DOMASEC framework to self-determination theory highlights the role of the individual as an active agent in their own development, and the fact that social-emotional competences develop over time, in interaction with significant others and changing contextual influences. It has been argued that SDT has the capacity to integrate different personality models, including the Big Five framework (Prentice et al., 2019; Ryan et al., 2019), and has the capacity to coordinate complex research findings concerning personality development, motivation, and wellbeing (Ryan et al., 2019). In particular, aspects of self-determination are relevant to understand the person as agent, as a motivated being making choices and planning their lives (McAdams and Olson, 2010), and can thus be helpful to inform strategies for building up social-emotional competences and the design of effective interventions. The development and maintenance of social-emotional competences can be facilitated if the needs for autonomy, relatedness and competence are met. For example, there is evidence to suggest that interventions aiming to support feelings of autonomy, relatedness and belonging can promote learning performance and persistence among students (Vansteenkiste et al., 2004; Skinner et al., 2009), or persistence in and adherence to physical exercise practice (Van den Berghe et al., 2014; Rodrigues et al., 2018). Linking the DOMASEC taxonomy to theories of self-determination and personality development thus facilitates recommendations for the design of developmentally appropriate interventions aiming to promote the development of social-emotional competences.

The DOMASEC taxonomy is designed to be broad enough to capture key aspects of different sets of constructs, to classify social-emotional competences studied across different disciplines, and to be specific enough to enable the grouping of social-emotional competences according to their core domains and their most central manifestations. Comparing the DOMASEC taxonomy with some of the frequently used frameworks used in the study of social-emotional learning, personality and character strengths illustrates its potential as an integrative tool. Future research should aim to link the DOMASEC framework to other classification tools with the objective to specify the core constructs and their different facets within the different cells of the grid in more detail, minimizing or eliminating overlap. Good examples of how this can be achieved can be found in the already mentioned online search tool (Explore SEL: http://exploresel.gse.harvard.edu/about/), or recent work mapping processes associated with executive functioning, a key feature of self-regulation, which also takes into account variations in expression across different developmental stages (Bailey et al., 2018). In addition, the taxonomy is useful to identify potential evidence gaps in current research. For example, relative many studies address issues related to the pre-cursors and long-term outcomes of self-control or self-regulation. There are, however, fewer studies examining the antecedents, development and outcomes associated with empathy or prosocial behavior, possibly due to the lack of strong measures for their assessment (Jones et al., 2016).

### Assessment of Social-Emotional Competences

The DOMASEC taxonomy also informs the assessment of social-emotional competences. Indeed, the grid structure could serve as a blueprint to facilitate test specification, or the cataloging of existing measurement tools (see also Bailey et al., 2018). There is a wide range of instruments, tapping into the different components and facets of social-emotional competences. Yet, while instruments to assess self-oriented competences such as self-regulation or self-concept are relatively common, there are fewer instruments to assess emotional competences that are, for example relevant for interaction with others, such as empathy (Halle and Darling-Churchill, 2016; Jones et al., 2016). A comprehensive assessment of social-emotional competences, however, should provide information about a range of different competences across different domains, involving different manifestations. Having information on multiple competences also enables the assessment of how these competences combine in individuals, giving insights into variations in competence profiles.

In developing new assessment instruments aiming for a more comprehensive appraisal of competence profiles, the standard requirements for a reliable and valid assessment should be fulfilled, as well as a number of practical considerations (Campbell et al., 2016; Jones et al., 2016): (a) measures should be developmentally appropriate in scope and content; moreover, they should enable researchers to assess the development of competences at earlier and later ages; (b) should be culturally appropriate; (c) should cover a comprehensive set of domains; (d) the administration of the assessment should not take too long, and should not put too much burden on the respondents; (e) to ensure consistency in administration there should not be too many training requirements for the administrator or observer; (f) there should be consideration for contextual issues of assessment, taking into account the setting of the assessment, as well as variations in expression across subgroups in the population or across different cultures.

Many available assessment instruments were developed for use in small-scale or specialized studies (Halle and Darling-Churchill, 2016). However, only a relative small number of these measures are suitable for administration in different types of studies, such as studies conducted across diverse populations and cultures, large scale surveys, studies focusing on social-emotional competences among very young children, or those aiming to assess continuity and change in social-emotional competences over time (Jones et al., 2016). For example, there is a scarcity of measures that are suitable for use with small children, instruments that cover a comprehensive range of competences, or enable the assessment of growth and development (Halle and Darling-Churchill, 2016).

Nonetheless, a key message is that social-emotional competences can be measured with relative precision and accuracy. Methods used to quantify the way individuals feel, think and behave across different situations have advanced considerably, in particular through the use of new technologies or involving multi-method multiple-informant approaches (Duckworth and Yeager, 2015; Abrahams et al., 2019). For example, computer-based problem scenarios (Rausch et al., 2019), interactive computer games (Day et al., 2019), or opportunistic measures derived from observing and coding the behaviors of individuals engaged in standardized assessment programs (Zamarro et al., 2018) can be used to balance the strengths and limitations of self-reports and direct assessments of social-emotional competences. Information about differences in behavior in different settings enables a better assessment of the multiple ways in which social-emotional competences manifest, how they develop over time, and how they vary across different contexts. Ideally, future assessments of social-emotional competences should provide information not only on single competences, but on a broader range of competences assessed across multiple domains and manifestations. A more comprehensive assessment would enable a more holistic understanding of how competences combine in individuals, and their relative and combined effect in shaping important life outcomes. Moreover, assessment tools that capture multiple components of social-emotional competences can be helpful to inform the planning of effective interventions, addressing specific strengths and deficits.

## Conclusion

Socio-emotional competences are critical for positive development and attainment across multiple domains, including education, employment, health and wellbeing (OECD, 2015). However, progress in empirical studies regarding the antecedents, correlates and long-term benefits of social-emotional competences is hampered by the lack of consensus about the number of key competences, how they are defined and operationalised. The conceptualization and measurement of social-emotional competences is not a straightforward task, because the term refers to a set of different capabilities. The proposed integrative taxonomy “DOMASEC” is understood as a framework supporting collaborative efforts to clearly delineate and distinguish core domains and manifestations of social-emotional competences and to facilitate conceptual clarity. Core domains include intrapersonal, interpersonal and task-oriented competencies, which are manifested in associated feelings, cognitions and behaviors. The DOMASEC typology helps to better organize the multitude of terms and definitions used, and to guide the conceptualization and operationalisation of social-emotional competences and their various facets. Providing a bridge between existing frameworks of social and emotional learning, personality traits (such as the Big Five), and the 21st century skill clusters the DOMASEC framework aims to generate a new dialogue between interlinked yet till now separated strands of investigation and to achieve a much needed consensus. Moreover, linking the DOMASEC specification of core domains to self-determination theories highlights the role of the individual as an active agent in their own development, and the fact that social-emotional competences develop over time, in interaction with significant others and changing contextual influences. This in turn, facilitates recommendations for the design of developmentally appropriate interventions aiming to promote the development of social-emotional competences.

It is hoped that the proposed taxonomy serves to connect different approaches regarding conceptualization and measurement, and hopefully bring about a consensus regarding the specification and delineation of core socio-emotional competencies and their assessment. Future work should focus in more detail on the specification of the different facets of socio-emotional competences, their comprehensive assessment across cultures, and review variations in the manifestation of distinct socio-emotional competences over time to reflect their formation, growth and possible changes.

## Ethics Statement

Ethical review and approval was not required for the study on human participants in accordance with the local legislation and institutional requirements. Written informed consent for participation was not required for this study in accordance with the national legislation and the institutional requirements.

## Author Contributions

IS conceptualized, drafted, and edited the focused review.

## Conflict of Interest

The author declares that the research was conducted in the absence of any commercial or financial relationships that could be construed as a potential conflict of interest.

## References

[B1] Abrahams L. Pancorbo G. Primi R. Santos D. Kyllonen P. John O. P.. (2019). Social-emotional skill assessment in children and adolescents: advances and challenges in personality, clinical, and educational contexts. Psychol. Assess. 31, 460–473. 10.1037/pas0000591 30869960

[B2] Achenbach T. M. (2019). International findings with the Achenbach System of Empirically Based Assessment (ASEBA): applications to clinical services, research, and training. Child Adolesc.Psychiatry Ment. Health 13. 10.1186/s13034-019-0291-2 31312253 PMC6610912

[B3] Bailey R. Barnes S. P. Park C. Sokolovic N. Jones S. M. (2018). Executive Function Mapping Project Measures Compendium: A Resource for Selecting Measures Related to Executive Function and Other Regulation-Related Skills in Early Childhood. OPRE Report # 2018-59. Retrieved from Washington, DC.

[B4] Bailey R. Meland E. A. Brion-Meisels G. Jones S. M. (2019). Getting developmental science back into schools: can what we know about self-regulation help change how we think about “no excuses”? Front. Psychol. 10:1885. 10.3389/fpsyg.2019.01885 31496971 PMC6712083

[B5] Berg J. Osher D. Same M. R. Nolan E. Benson D. Jacobs N. (2017). Identifying, defining, and Measuring Social and Emotional Competencies. Retrieved from American Institutes for Research. Availble online at: https://www.air.org/resource/identifying-defining-and-measuring-social-and-emotional-competencies (accessed February 26, 2021).

[B6] Blair C. (2002). School readiness–integrating cognition and emotion in a neurobiological conceptualization of children's functioning at school entry. Am. Psychol. 57, 111–127. 10.1037/0003-066X.57.2.111 11899554

[B7] Bleidorn W. Hopwood C. J. Lucas R. E. (2018). Life events and personality trait change. J. Personal. 86, 83–96. 10.1111/jopy.12286 27716921

[B8] Booth A. Hennessy E. Doyle O. (2018). Self-regulation: learning across disciplines. J. Child Family Stud. 27, 3767–3781. 10.1007/s10826-018-1202-5

[B9] Borghans L. Duckworth A. L. Heckman J. ter Weel B. J. (2008). The economics and psychology of personality traits. J. Hum. Resour. 43, 972–1059. 10.1353/jhr.2008.0017

[B10] Boyatzis R. E. (2018). The behavioral level of emotional intelligence and its measurement. Front. Psychol. 9:1438. 10.3389/fpsyg.2018.01438 30150958 PMC6099111

[B11] Bronfenbrenner U. Morris P. A. (2006). "The bioecological model of human development,” in Theoretical Models of Human Development. Handbook of Child Psychology, Vol. 1, 6th Edn, ed Lerner R. M. (Hoboken, NJ: Wiley), 793–828.

[B12] Campbell S. B. Denham S. A. Howarth G. Z. Jones S. M. Whittaker J. V. Williford A. P.. (2016). Commentary on the review of measures of early childhood social and emotional development: conceptualization, critique, and recommendations. J. Appl. Dev. Psychol. 45, 19–41. 10.1016/j.appdev.2016.01.008

[B13] Caspi A. Roberts B. W. Shiner R. L. (2005). Personality development: stability and change. Annu. Rev. Psychol. 56, 453–484. 10.1146/annurev.psych.55.090902.141913 15709943

[B14] Cheung F. M. Fan W. Cheung S. F. Leung K. (2008). Standardization of the cross-cultural [Chinese] personality assessment inventory for adolescents in Hong Kong: a combined emic-etic approach to personality assessment. Acta Psychol. Sin. 40, 839–852. 10.3724/SP.J.1041.2008.01639

[B15] Cheung F. M. van de Vijver F. J. R. Leong F. T. L. (2011). Toward a new approach to the study of personality in culture. Am. Psychol. 66, 593–603. 10.1037/a0022389 21261408

[B16] Costa P. T. McCrae R. R. Lockenhoff C. E. (2019). Personality across the life span. Annu. Rev. Psychol. 70, 423–448. 10.1146/annurev-psych-010418-103244 30231002

[B17] Damian R. I. Spengler M. Sutu A. Roberts B. W. (2019). Sixteen going on sixty-six: a longitudinal study of personality stability and change across 50 years. J. Personal. Soc. Psychol. 117, 674–695. 10.1037/pspp0000210 30113194

[B18] Day J. Freiberg K. Hayes A. Homel R. (2019). Towards scalable, integrative assessment of children's self-regulatory capabilities: new applications of digital technology. Clin. Child Fam. Psychol. Rev. 22, 90–103. 10.1007/s10567-019-00282-4 30737606

[B19] De Fruyt F. Wille B. John O. P. (2015). Employability in the 21st century: complex (interactive) problem solving and other essential skills. Ind. Organ. Psychol. Perspect. Sci. Pract. 8, 276–U189. 10.1017/iop.2015.33

[B20] De Raad B. Barelds D. P. H. Levert E. Ostendorf F. Mlacic B. Di Blas L.. (2010). Only three factors of personality description are fully replicable across languages: a comparison of 14 trait taxonomies. J. Personal. Soc. Psychol. 98, 160–173. 10.1037/a0017184 20053040

[B21] Domitrovich C. E. Durlak J. A. Staley K. C. Weissberg R. P. (2017). Social-emotional competence: an essential factor for promoting positive adjustment and reducing risk in school children. Child Dev. 88, 408–416. 10.1111/cdev.12739 28213889

[B22] Duckworth A. L. Peterson C. Matthews M. D. Kelly D. R. (2007). Grit: perseverance and passion for long-term goals. J. Personal. Soc. Psychol. 92, 1087–1101. 10.1037/0022-3514.92.6.1087 17547490

[B23] Duckworth A. L. Yeager D. S. (2015). Measurement matters: assessing personal qualities other than cognitive ability for educational purposes. Educ. Res. 44, 237–251. 10.3102/0013189X15584327 27134288 PMC4849415

[B24] Durlak J. A. Domitrovich C. E. Weissberg R. P. Gulotta T. P. (eds.). (2015). Handbook of Social and Emotional Learning: Research and Practice. New York, NY: Guildford Press.

[B25] Durlak J. A. Weissberg R. P. Dymnicki A. B. Taylor R. D. Schellinger K. B. (2011). The impact of enhancing students' social and emotional learning: a meta-analysis of school- based universal interventions. Child Dev. 82, 405–432. 10.1111/j.1467-8624.2010.01564.x 21291449

[B26] Elias M. J. Haynes N. M. (2008). social competence, social support, and academic achievement in minority, low-income, urban elementary school children. Sch. Psychol. Q. 23, 474–495. 10.1037/1045-3830.23.4.474

[B27] Goodman R. Ford T. Simmons H. Gatward R. Meltzer H. (2000). Using the Strengths and Difficulties Questionnaire (SDQ) to screen for child psychiatric disorders in a community sample. Br. J. Psychiatry 177, 534–539. 10.1192/bjp.177.6.534 11102329

[B28] Gresham F. Elliott S. Metallo S. Byrd S. Wilson E. Erickson M.. (2020). Psychometric fundamentals of the social skills improvement system: social-emotional learning edition rating forms. Assess. Effect. Interv. 45, 194–209. 10.1177/1534508418808598

[B29] Gutman L. M. Schoon I. (2016). “A synthesis of causal evidence linking non-cognitive skills to later outcomes for children and adolescents,” in Non-Cognitive Skills and Factors in Educational Attainment, Vol. 9, eds Khine M. S. Areepattamannil S. (Rotterdam: Sense Publishers), 171–198. 10.1007/978-94-6300-591-3_9

[B30] Halle T. G. Darling-Churchill K. E. (2016). Review of measures of social and emotional development. J. Appl. Dev. Psychol. 45, 8–18. 10.1016/j.appdev.2016.02.00328158522

[B31] Heckman J. J. Kautz T. (2012). Hard evidence on soft skills. Labour Econ. 19, 451–464. 10.1016/j.labeco.2012.05.014 23559694 PMC3612993

[B32] John O. P. Naumann L. P. Soto C. J. (2008). “Paradigm shift to the integrative big-five trait taxonomy: history, measurement, and conceptual issues.” in Handbook of Personality: Theory and Research, eds John O. P. Robins R. W. Pervin L. A. (New York, NY: Guilford Press), 114–158.

[B33] Jones S. M. McGarrah M. W. Kahn J. (2019). Social and emotional learning: a principled science of human development in context. Educ. Psychol. 54, 129–143. 10.1080/00461520.2019.1625776

[B34] Jones S. M. Zaslow M. Darling-Churchill K. E. Halle T. G. (2016). Assessing early childhood social and emotional development: key conceptual and measurement issues. J. Appl. Dev. Psychol. 45, 42–48. 10.1016/j.appdev.2016.02.008

[B35] Liu A. (2019). Can non-cognitive skills compensate for background disadvantage? The moderation of non-cognitive skills on family socioeconomic status and achievement during early childhood and early adolescence. Soc. Sci. Res. 83:102306. 10.1016/j.ssresearch.2019.04.019 31422837

[B36] Malti T. Noam G. G. (2016). Social-emotional development: from theory to practice. Eur. J. Dev. Psychol. 13, 652–665. 10.1080/17405629.2016.1196178

[B37] Mantz L. S. Bear G. G. Yang C. Y. Harris A. (2018). The Delaware Social-Emotional Competency Scale (DSECS-S): evidence of validity and reliability. Child Indic. Res. 11, 137–157. 10.1007/s12187-016-9427-6

[B38] McAdams D. P. Olson B. D. (2010). Personality development: continuity and change over the life course. Annu. Rev. Psychol. 61, 517–542. 10.1146/annurev.psych.093008.100507 19534589

[B39] McCrae R. R. Terracciano A. Personal Profiles Cultures Project (2005). Universal features of personality traits from the observer's perspective: data from 50 cultures. J. Personal. Soc. Psychol. 88, 547–561. 10.1037/0022-3514.88.3.547 15740445

[B40] McGrath R. E. Greenberg M. J. Hall-Simmonds A. (2018). Scarecrow, tin woodsman, and cowardly lion: the three-factor model of virtue. J. Posit. Psychol. 13, 373–392. 10.1080/17439760.2017.1326518

[B41] McGrath R. E. Hall-Simmonds A. Goldberg L. R. (2020). are measures of character and personality distinct? Evidence from observed-score and true-score analyses. Assessment 27, 117–135. 10.1177/1073191117738047 29073771 PMC5878981

[B42] Moffitt T. E. Arseneault L. Belsky D. Dickson N. Hancox R. J. Harrington H.. (2011). A gradient of childhood self-control predicts health, wealth, and public safety. Proc. Natl. Acad. Sci. U.S.A. 108, 2693–2698. 10.1073/pnas.1010076108 21262822 PMC3041102

[B43] Montroy J. J. Bowles R. P. Skibbe L. E. McClelland M. M. Morrison F. J. (2016). The development of self-regulation across early childhood. Dev. Psychol., 52, 1744–1762. 10.1037/dev0000159 27709999 PMC5123795

[B44] National Research Council (2012). Education for Life and Work: Developing Transferable Knowledge and Skills in the 21st century. Retrieved from Washington, DC: The National Academies Press. Available online at: https://www.nap.edu/download/13398 (accessed February 26, 2021).

[B45] OECD (2015). Skills for Social Progress. The Power of Social And Emotional Skills. Available online at: http://www.oecd-ilibrary.org/education/skills-for-social-progress_9789264226159-en (accessed February 26, 2021).

[B46] Osher D. Kidron Y. Brackett M. Dymnicki A. Jones S. Weissberg R. P. (2016). Advancing the science and practice of social and emotional learning: looking back and moving forward. Rev. Res. Educ. 40, 644–681. 10.3102/0091732X16673595

[B47] Pellegrino J. W. Hilton M. L. (Eds.). (2012). Education for Life And Work: Developing Transferable Knowledge and Skills in the 21st Century. Washington, DC: The National Academic Press. Available online at: https://hewlett.org/wp-content/uploads/2016/08/Education_for_Life_and_Work.pdf.

[B48] Prentice M. Jayawickreme E. Fleeson W. (2019). Integrating whole trait theory and self-determination theory. J. Personal. 87, 56–69. 10.1111/jopy.12417 29999534

[B49] Rausch A. Kogler K. Seifried J. (2019). Validation of Embedded Experience Sampling (EES) for measuring non-cognitive facets of problem-solving competence in scenario-based assessments. Front. Psychol. 10:1200. 10.3389/fpsyg.2019.01200 31178807 PMC6542984

[B50] Roberts B. W. Luo J. Briley D. A. Chow P. I. Su R. Hill P. L. (2017). A systematic review of personality trait change through intervention. Psychol. Bull. 143:117. 10.1037/bul0000088 28054797

[B51] Rodrigues F. Bento T. Cid L. Neiva H. P. Teixeira D. Moutao J.. (2018). Can interpersonal behavior influence the persistence and adherence to physical exercise practice in adults? a systematic review. Front. Psychol. 9:2141. 10.3389/fpsyg.2018.02141 30459690 PMC6232376

[B52] Ryan R. Deci E. (2017). Self-Determination Theory: Basic Psychological Needs in Motivation, Development, and Wellness. New York, NY: Guilford Press. 10.1521/978.14625/28806

[B53] Ryan R. M. Soenens B. Vansteenkiste M. (2019). Reflections on self-determination theory as an organizing framework for personality psychology: interfaces, integrations, issues, and unfinished business. J. Personal. 87, 115–145. 10.1111/jopy.12440 30325499

[B54] Rychen D. S. Salganik L.H. (eds.). (2003). Key Competencies for a Successful Life and a Well-Functioning Society. Göttingen Hogrefe and Huber.

[B55] Sameroff A. J. (2010). A unified theory of development: a dialectic integration of nature and nurture. Child Dev. 81, 6–22. Retrieved from *< Go to ISI>://WOS:000274308300001* 10.1111/j.1467-8624.2009.01378.x 20331651

[B56] Schoon I. Lyons-Amos M. (2017). A socio-ecological model of agency: the role of structure and agency in shaping education and employment transitions in England. Longitud. Life Course Stud., 8, 35–56. 10.14301/llcs.v8i1.404

[B57] Skinner E. A. Kindermann T. A. Furrer C. J. (2009). A motivational perspective on engagement and disaffection conceptualization and assessment of children's behavioral and emotional participation in academic activities in the classroom. Educ. Psychol. Meas. 69, 493–525. 10.1177/0013164408323233

[B58] Soto C. J. Napolitano C. M. Roberts B. W. (2020). Taking skills seriously: toward an integrative model and agenda for social, emotional, and behavioral skills. Curr. Dir. Psychol. Sci. 10.1177/0963721420978613

[B59] Van den Berghe L. Vansteenkiste M. Cardon G. Kirk D. Haerens L. (2014). Research on self-determination in physical education: key findings and proposals for future research. Phys. Educ. Sport Pedag. 19, 97–121. 10.1080/17408989.2012.732563

[B60] Vansteenkiste M. Simons J. Lens W. Sheldon K. A. Deci E. L. (2004). Motivating learning, performance, and persistence: the synergistic effects of intrinsic goal contents and autonomy-supportive contexts. J. Personal. Soc. Psychol. 87, 246–260. 10.1037/0022-3514.87.2.246 15301630

[B61] Wilt J. Revelle W. (2015). Affect, behaviour, cognition and desire in the big five: an analysis of item content and structure. Eur. J. Personal. 29, 478–497. 10.1002/per.2002 26279606 PMC4532350

[B62] Zamarro G. Cheng A. Shakeel M. D. Hitt C. (2018). Comparing and validating measures of non-cognitive traits: performance task measures and self-reports from a nationally representative internet panel. J. Behav. Exp. Econ. 72, 51–60. 10.1016/j.socec.2017.11.005

